# Physical activity and treatment adherence in patients with mental disorders: a randomized controlled trial

**DOI:** 10.1192/j.eurpsy.2024.177

**Published:** 2024-08-27

**Authors:** R. Silvestro, V. Dello Stritto, P. Catapano, M. Di Vincenzo, B. Della Rocca, M. V. Lapadula, C. Toni, R. Bello, M. Luciano, G. Sampogna, A. Fiorillo

**Affiliations:** Psychiatry, Università degli studi della Campania “Luigi Vanvitelli”, Naples, Italy

## Abstract

**Introduction:**

Lack of adherence to pharmacological treatment is considered a multifactorial phenomenon, remarkably frequent in clinical practice. Non-adherence is associated with increased number of relapses, poor clinical and functional outcomes, and worsening of patient health status, with a resulting increase in healthcare costs, particularly in people with severe mental disorders (SMD). Treatment adherence rates remain extremely low, highlighting the need to develop innovative and integrated strategies; one of these is represented by the promotion of healthy lifestyle behaviours, including regular physical activity.

**Objectives:**

The aim of this study is to assess how the rates of treatment adherence vary in patients with SMD after receiving a psychosocial intervention, focusing on the positive relationship between treatment adherence and physical activity.

**Methods:**

LIFESTYLE is a randomized controlled trial comparing the efficacy of a structured psychosocial lifestyle intervention involving moderate physical activity exercises over a brief psychoeducational intervention. Levels of physical activity was assessed thorough the IPAQ scale, while treatment adherence was evaluated by the Morisky Medication Adherence Scale (MMAS).

**Results:**

The sample includes 401 patients, with a mean duration of illness was 16.3 (±17.8) years. All patients were receiving a pharmacological drug treatment; in particular, 59.6% (N=239) were treated with a second-generation antipsychotic and 54.9% (N=220) with a mood stabilizer. Our results show that moderate physical activity improves rates of treatment adherence. After 6 months, adherence to treatment increased from 35.8% at baseline to 47.6% at baseline in the experimental group, along with improvement in clinical health parameters (reduction in BMI, weight, and metabolic parameters). Another significant inverse correlation was found between adherence and quality of life (Rho di Person: -.140, p<.005). Furthermore, this study indicates that having a diagnosis of major depression, a better cognitive functioning, a shorter duration of illness and contact time with the local mental health centre are factors that positively influence treatment adherence. Remarkably, treatment adherence was not influenced by symptom severity and type of pharmacological treatment.

**Conclusions:**

Moderate physical activity can represent a valid strategy to increase treatment adherence in patients with SMD. Therefore, promoting physical activity exercises in our clinical practice may be associated with better outcomes. However, further studies that evaluate patients with acute mental disorders are needed.

**Disclosure of Interest:**

None Declared

